# Stereotactic body radiotherapy extends the clinical benefit of PD-1 inhibitors in refractory recurrent/metastatic nasopharyngeal carcinoma

**DOI:** 10.1186/s13014-022-02073-8

**Published:** 2022-07-05

**Authors:** Jing Lin, Qiaojuan Guo, Zengqing Guo, Tianzhu Lu, Gang Chen, Shaojun Lin, Mei Chen, Chuanben Chen, Jianping Lu, Jingfeng Zong, Lina Tang, Yu Chen, Jianji Pan

**Affiliations:** 1 Department of Medical Oncology, Fujian Medical University Cancer Hospital and Fujian Cancer Hospital, Fuzhou, 350014 Fujian Province China; 2 Cancer Bio-Immunotherapy Center, Fujian Medical University Cancer Hospital & Fujian Cancer Hospital, No. 420 Fuma Road, Fuzhou, 350014 Fujian Province China; 3 Department of Radiation Oncology, Fujian Medical University Cancer Hospital & Fujian Cancer Hospital, Fuzhou, 350014 Fujian Province China; 4 Fujian Provincial Key Laboratory of Translational Cancer Medicine, Fuzhou, Fujian China; 5 Department of Pathology, Fujian Medical University Cancer Hospital & Fujian Cancer Hospita, Fuzhou, Fujian Province China; 6 Department of Ultrasound, Fujian Medical University Cancer Hospital & Fujian Cancer Hospital, Fuzhou, Fujian Province China; 7 College of Chemistry, Fuzhou University, Fuzhou, China

**Keywords:** Stereotactic body radiotherapy, PD-1, Immunotherapy, Nasopharyngeal carcinoma

## Abstract

**Purpose:**

Emerging evidence shows that immune checkpoint inhibitors lead to durable responses in a variety of cancers, including nasopharyngeal carcinoma (NPC), however, combination approaches (i.e., stereotactic body radiation therapy, SBRT) are required to extend this benefit beyond a subset of patients. This study retrospectively evaluated eight recurrent/metastatic NPC patients, to investigate how radiation could potentiate PD-1 checkpoint inhibition therapy.

**Methods:**

Between September 2016 and July 2017, eight consecutive cases with histologically confirmed PDL1-positive status, for which prior standard therapy had been ineffective (five patients), were treated at our institution and Macao Clinics and two patients had disease progression within 6 months of completion of definitive chemoradiation, or one patient refused to receive chemoradiotherapy. All received PD-1 inhibitors first, seven of them accepted SBRT with an unmodified PD-1 inhibitors regimen after first evaluation as they were unresponsive to PD-1 inhibitors alone. Treatment was discontinued as long as patients were experiencing a clinical benefit in the opinion of the physicians and at least five cycles were given before stoppage.

**Results:**

Median follow-up time was 56.7 months. The confirmed objective response rate based on RECIST-v1.1 at first evaluation was 12.5% (1/8). For the seven cases who received SBRT, six of them experience an objective response (6/7, 85.7%) after SBRT. Only one patient showed rapid progress and die within 95 days after the initiation of SBRT intervention. Three patients who did not have all lesions exposed to irradiation were available to evaluate the incidence of an abscopal effect, however, it did not occur as expected. Median PFS and OS for the seven patients were 8.0 and 30.8 months after SBRT intervention, respectively. Two-year OS as indicated was 71.0%.

**Conclusions:**

PD-1 inhibitors combined with SBRT demonstrated promising antitumor activity in patients with PD-L1 positive RM-NPC. Patients may benefit from continue immunotherapy beyond disease progression when SBRT was introduced.

## Background

Recurrent/metastatic nasopharyngeal carcinoma (RM-NPC) remains a disease associated with few therapeutic options, for which standard-of-care is platinum-containing combination therapy [1]. Although the response rates to this treatment modality has been reported to be higher than 50%, the duration of the response and survival time are limited [2]. This is especially true for patients who relapse with distant metastasis, with reported median survival times ranging from only 5 to 11 months [3–6]. For patients who have progressed beyond a first-line setting with platinum-refractory RM-NPC, there are no standard treatment options. Salvage second-line chemotherapy and targeted drugs only produce moderate antitumor activity as second-line or later treatment in this setting [6–9]. Given these outcomes, the need for a more effective therapy for patients with incurable NPC is clear.

Progress made in the field of immunotherapy (i.e. PD-1/PDL1 checkpoint inhibition) has led to promising breakthroughs in treating various solid malignancies [10–12]. Exploration of immunotherapy with PD-1/PDL1 checkpoint blockade in RM-NPC patients beyond first-line treatment has been reported in clinical trials with relatively small samples [13–15], with all patients having received monotherapy with pembrolizumab, nivolumab, or camrelizumab. Although encouraging response rates of 19% ~ 34% were reported in this subgroup of patients, there still remain a substantial number of patients that do not respond and fail to have a long-lasting clinical benefit. Apart from identification of biomarkers to select the patients who are likely to respond to PD-1 blockade beforehand, another potential method for improving the response rate is combining with traditional oncological interventions, one of which is radiotherapy [16–20].

Published data has revealed that the combination of radiotherapy with anti-PD1 treatment ca lead to a synergistic effect, thereby enhancing response rates [17, 21–27]. Radiation-induced immune responses might be dose-dependent, using radiation doses in the ‘ablative’ range can not only effectively destroy tumor cells directly, but might also encourage these SABR-killed cells to function as a vaccine in situ [17, 28–31]. In addition, radiation can re-program the tumor stromal microenvironment against the immune evasion mechanisms of cancer [32]. There is a report from Desideri et al. that there are different responses to combined therapy with nivolumab when using ablative vs. palliative RT [33]. Thus, there was the concept of “ISABR” (immunotherapy and stereotactic ablative radiotherapy) proposed by Prof. Chang [17]. This concept has been clinically reported for multiple diseases, including case reports of lung cancer and melanoma [34, 35]. However, this combination has not been reported in RM-NPC patients.

To further evaluate the efficacy of PD-1 inhibitors and its combination with SBRT in recurrent/metastatic NPC patients, we report our experience in eight RM-NPC patients who were treated in Macao Clinics and our institution. Our results will shed light on the toxicity and potential anti-tumor activity of this combination.

## Methods

### Patients and pretreatment evaluation

This retrospective study was approved by the Research Ethics Committee of our institution. Eight NPC patients treated with PD-1 inhibitors, with or without radiotherapy, between October 2016 and August 2017 were included. They were all histologically confirmed with RM-NPC, for which prior standard therapy was ineffective (five patients), had disease progression within 6 months of completion of definitive chemo-radiotherapy (two patients), or refused to receive chemoradiotherapy (one patients). All of them were classified as WHO type II/III and has an Eastern Cooperative Oncology Group performance status (ECOG PS) of 0 ~ 2, and adequate organ function as determined by laboratory testing. As the PD-1 inhibitors were not yet available in Mainland China at that time, the PD-1 inhibitors were given at Macao Clinics, with regular follow-up, efficacy evaluation, and radiotherapy performed at our institution. None of them had received previous treatments that specifically targeted T-cell co-stimulation or checkpoint pathways.

### PD-1 inhibitors administration

Treatment programs were developed after full discussion in multidisciplinary panels including medical oncologists, immunologists, and radiation oncologists. Nivolumab (Opdivo, Bristol-Myers Squibb) and pembrolizumab (Keytruda, Merck & Co) were given at 3 mg/kg and 2 mg/kg intravenously once every 2 and 3 weeks, respectively. The selection of these two drugs was not protocolized and was used according to the required dosage and drug specifications, among which economic factors were fully taken into consideration. Due to financial limitations, most patients could not receive long-term PD-1 inhibitors administration. Treatment was discontinued as long as patients were experiencing clinical benefit in the opinion of the physicians and at least five cycles were given.

### Radiotherapy

The timing of SBRT intervention depends on the expression of PD-L1 and CD8 + tumor infiltrating lymphocytes (TIL), and the response to PD-1 inhibitors. Since all eight patients presented with PD-L1 expression and CD8 + TIL (adaptive resistance) [36], they were first treated by PD-1 inhibitors alone and SBRT was introduced only when patients did not response to PD-1 inhibitors.

SBRT was used to irradiate all the visible lesions or the main recurrent and/or metastatic lesions or progressive lesions, with a radiation dose of 25-36 Gy (5 ~ 6 Gy/5 ~ 6Fx). The setting of the tumor target area and radiation dose and the formulation of the radiotherapy plan were all conducted under the guidance of the chief radiotherapy oncologist (JJ Pan) in our hospital. The dose fractionation scheme was individualized based upon the specific condition of each case and the history of past treatment was taken into consideration as well.

The Gross Target Volume (GTV) was defined as visible tumors by combining iconographic and metabolic information for all or part of the lesions. No additional margins were added for microscopic spread of disease. The GTV was expanded with 2–5 mm to the Planning Target Volume (PTV) to account for organ motion and setup error according to localization of the metastasis. More details of radiotherapy are indicated in Table 1.

**Table 1:** Patient characteristics and treatment details

Patient No	Sex	Age	ECOG PS	Recurrent/metastasis status	oligometastasis	Number of previous lines of therapy
1	M	28	2	Liver and multiple bone metastasis (thoracic and lumbar vertebra, pelvis, sternum, bilateral femur, bilateral ribs, right humerus)	No	2
2	M	52	0	Multiple liver metastasis	Yes	2
3	F	53	1	Local recurrence, multiple sites in liver, hilar lymph node, left scapula	Yes	1 (refused to receive chemotherapy)
4	M	44	1	Metastasis in liver and in right axillary lymph nodes	Yes	2
5	M	41	0	Multiple metastasis in liver and bone (lumbar vertebra)		PD within 6 months
6	M	56	1	Multiple metastasis (right 6th rib, right axillary lymph nodes and multiple lung nodules)	Yes	4
7	M	48	1	Regional refractory in bilateral neck; liver and multiple bone metastasis (sternum, thoracic and lumbar vertebra)	No	PD within 3 months
8	M	39	2	Local and regional relapse in nasopharynx and bilateral neck	NA	2

F, female; M, male; ECOG PS, Eastern Cooperative Oncology Group performance status; PD, progressive disease

### Immunohistochemical analysis for PD-L1, CD8, PD-1, CD3

Immunohistochemistry for CD3, CD8, PD-L1, and PD-1 were performed with CONFIRM anti-CD3 (2GV6) antibody (VENTANA, 790–4341), (790–4460) CONFIRM CD8 (SP57) antibody (VENTANA, 790–4460), PD-L1 (SP142) Assay (VENTANA, 740–4859), and anti-PD-1 antibody (MXB Biotechnologies, MAB-0743) using an automated slide stainer (BenchMark XT, VENTANA Medical Systems, Tucson, AZ, USA), the antibodies were directly added into the stainer without dilution.. The tissue sections were screened at low magnification (× 100) and measured at × 400 magnification.

### Follow-up and statistical analysis

The overall evaluation of the treatment response was based on RECIST version 1.1. Follow-up evaluations for all patients were performed every 2–3 cycles of PD-1 inhibitor immunotherapy. Each follow-up included a complete history and physical examination, as well as basic serum chemistry, plasma EB-DNA, and imaging examination (CT and/or MRI and/or PET-CT) of the corresponding lesions. Toxicity during treatment was graded according to the CTCAE v.4.0.

The best objective response rate (ORR), overall survival (OS), and progression free survival (PFS) were evaluated as well. The OS was recorded from the day of treatment with PD-1 inhibitors to the date of death or last follow-up. The PFS was recorded from the day of SBRT intervention to disease progression or death. Kaplan–Meier survival analyses were used to estimate OS. Statistical analyses were performed using SPSS software, version 18.0 (SPSS, Inc., Chicago, IL, USA).

## Results

### Patient characteristics

Eight patients who were R/M NPC after the first treatment were included in this case series. The median follow-up time was 56.7 months (range: 6.1–61.1 months), and three patients were alive during the last follow-up. Baseline patient characteristics are presented in Table 1. The median age was 46 years (range: 28–56 years), with one female patient. Five patients (87.5%) received at least two lines of chemotherapy. Two patients progressed in 6 months after initial treatment. One patient (12.5%) with multiple metastatic NPC refused to receive chemoradiotherapy. Two patients (25%) had an ECOG performance status of 2, four (50%) were ECOG 1, and one (25%) was ECOG 0. According to the classification of PD-L1 and CD8 + TIL, all patients showed adaptive resistance (PD-L1 expression and CD8 + TIL).

#### Anti-tumor activity of PD-1 inhibitors alone

**Fig. 1:**
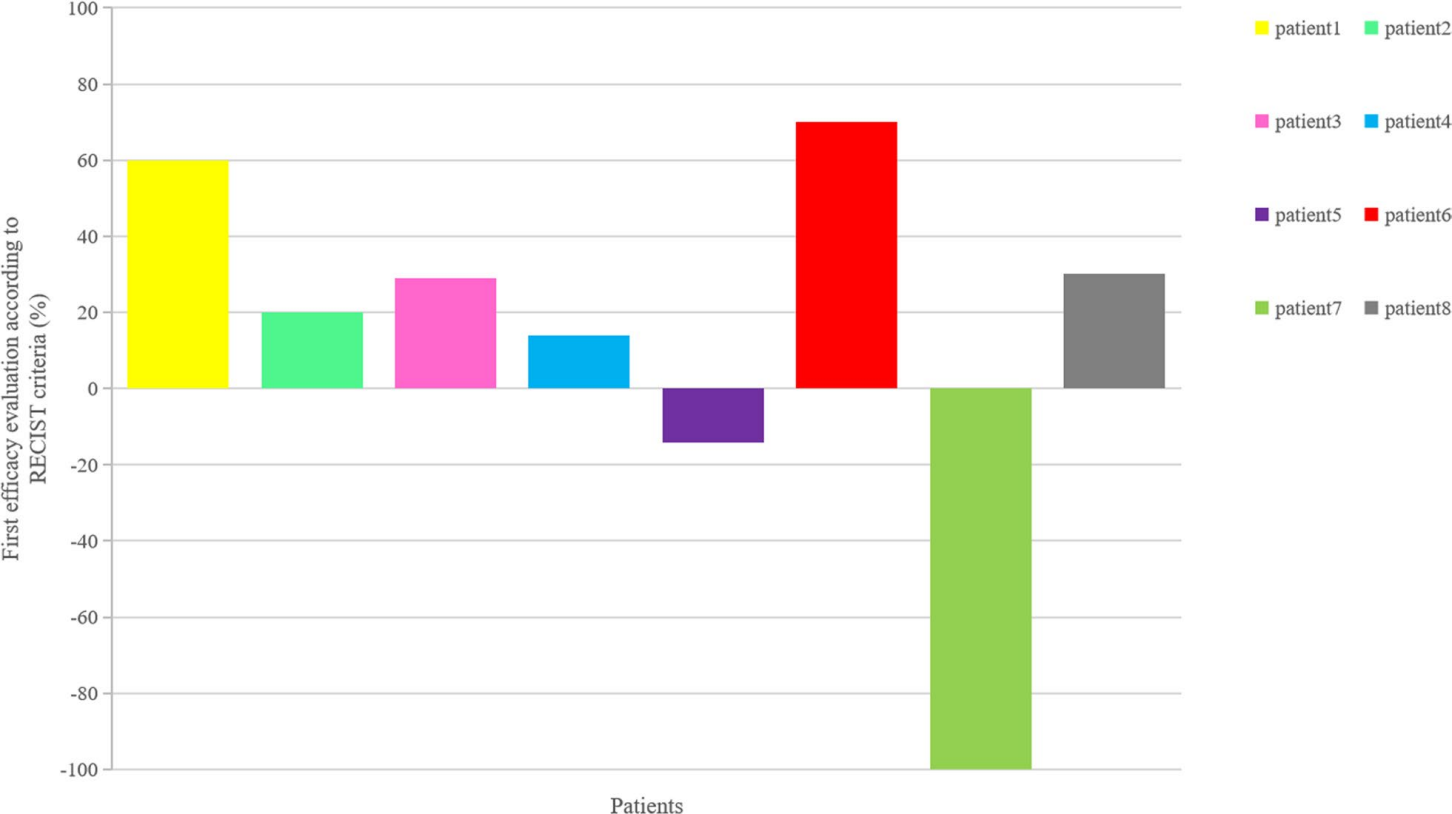
Efficacy evaluation of 2-3 cycles of PD1 inhibitors in patients with Nasopharyngeal Carcinoma

Since all the eight patients presented with a uniform immunophenotype of adaptive resistance, PD-1 inhibitors were given to the whole cohort at the beginning. After 2–3 cycles of PD-1 inhibitors, all patients were evaluated according to RECIST 1.1 and one patient had a CR, two had SD, and the remaining five had PD. The confirmed ORR and disease control rate (DCR) at 3 cycles of PD-1 inhibitors alone was 12.5% (1 in 8) and 37.5% (3 in 8), respectively (Fig. 1). Notably, patient 7#, who achieved a CR after just 3 cycles of PD-1 inhibitor, presented with new distant metastatic lesions 18 months later. After a panel discussion he received another 3 cycles of PD-1 inhibitor and again achieved a CR. What is worth mentioning is that this status continued up to August 27, 2020.

#### SBRT extends the clinical benefit of PD-1 inhibitors

As demonstrated above, only one patient obtained an objective response at first evaluation. The remaining seven patients were then given SBRT (25 ~ 36 Gy/5 ~ 6Fx, six fraction-regimen, typically 6 × 5 Gy, were preferred, detailed in Table 2) to irradiate all visible lesions or main lesions or only progressive lesions. The aim was to improve the treatment response to PD-1 inhibitors. It is noteworthy that radiotherapy did not interfere with the administration of PD-1 inhibitors. Second evaluation based on RECIST 1.1 was performed after SBRT. The ORR of these seven cases was 85.7% (6/7 cases), including two CR (28.6%, 2/7) and four PR (57.1%, 4/7). The remaining patient (patient #1) progressed during the treatment course and died within 95 days after the initiation of SBRT).

**Table 2:** The detail treatment and therapeutic evaluation of 8 patients

ID	PD 1 inhibitors	SBRT		Therapeutic evaluation		
		Dose	Radiation sites	PD-1inhibitor alone	Radiated lesions	Non-Radiated lesions
1	Nivolumab (5 cycles)	25 Gy/5Fx	Progressive lesions	PD	PR	PD
2	Pembroizumab (6 cycles)	30 Gy/6Fx	All visible lesions	PD	CR	NA
3	Nivolumab (5 cycles)	30 Gy/6Fx	Progressive lesions	PD	CR	Unevaluable
4	Nivolumab (14 cycles)	36 Gy/6Fx	Main lesions	SD	PR	SD
5	Nivolumab (11 cycles)	30 Gy/6Fx	Main lesions	SD	PR	SD
6	Nivolumab (6 cycles)	30 Gy/6Fx	All visible lesions	PD	CR	NA
7	Nivolumab (6 cycles)	No	NA	CR	NA	NA
8	Nivolumab (9 cycles)	30 Gy/6Fx	Progressive lesions	PD	PR	Unevaluable

CR: complete response; PR: partial response; SD: stable disease; PD: progressive disease; NA: not assessed

#### Evaluation of the abscopal effect of SBRT

**Fig. 2:**
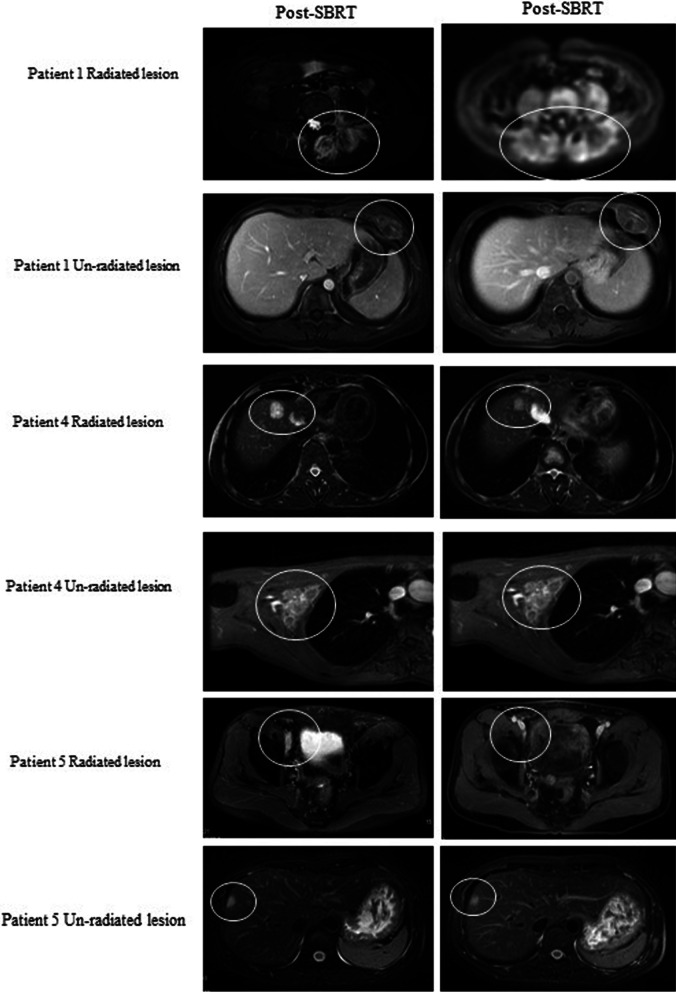
The dynamic imaging responses of all irradiated and non-irradiated lesions (patients # 1, # 4 and #5)

To assess the abscopal effect of SBRT an objective response of irradiated and non-irradiated lesions was separately evaluated according to the criteria of RECIST 1.1. Of the seven patients who underwent SBRT, three and four patients eventually achieved a CR and PR of the irradiated lesions, respectively. Two of them received irradiation to all visible lesions, with no lesions being available to evaluate an abscopal effect. For the other five cases who underwent SBRT directed against progressive lesions or major lesions, only three patients were suitable for assessing the abscopal effect (patients #1, #4, and #5). None of the evaluable non-irradiation lesions showed an objective response, which meant that no abscopal effects was observed in our cohort. The dynamic imaging responses of all irradiated and non-irradiated lesions are shown in Fig. 2.

### Survival analysis

**Fig. 3:**
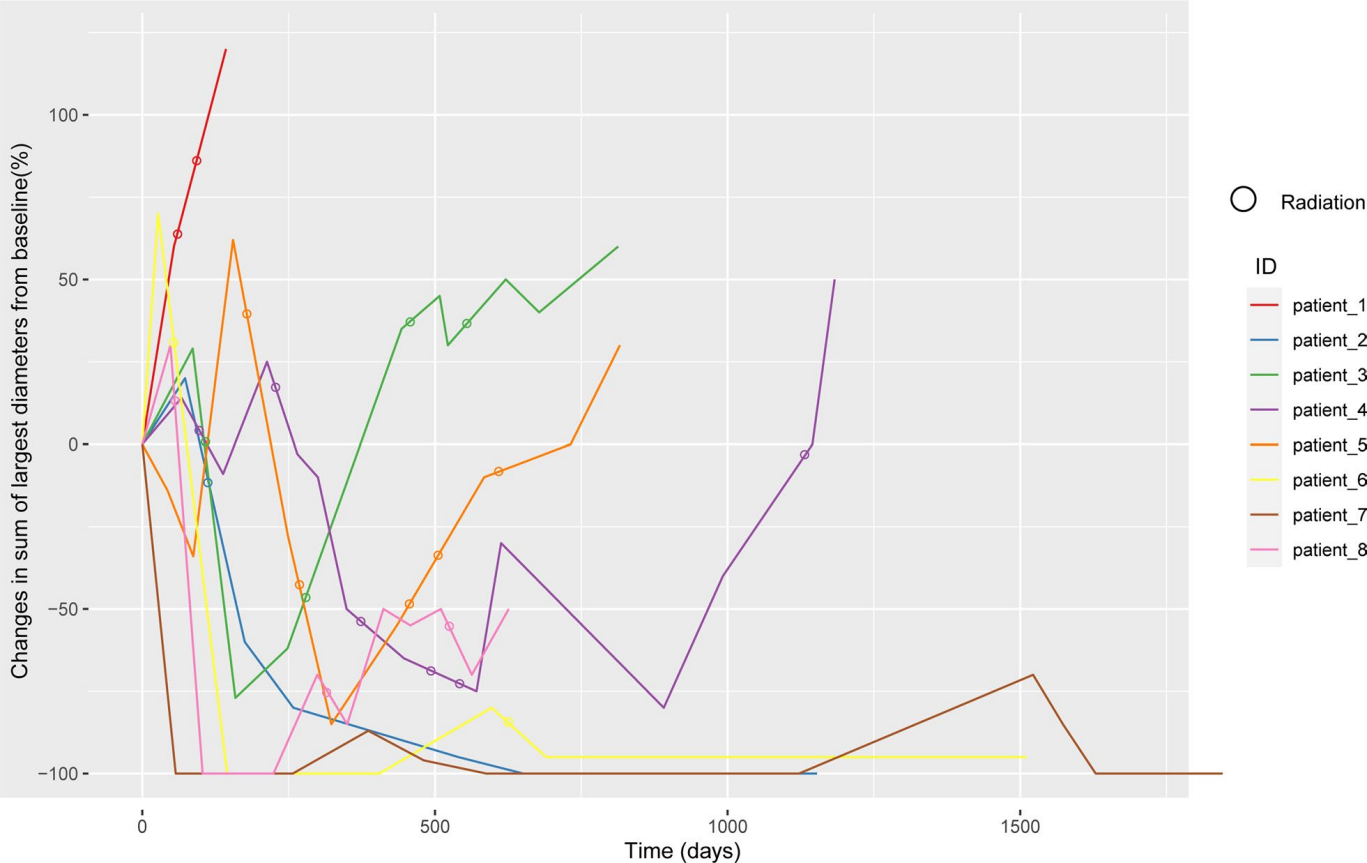
Tumor load and radiotherapy time during follow-up of immunotherapy and stereotactic ablative radiotherapy patients

**Fig. 4:**
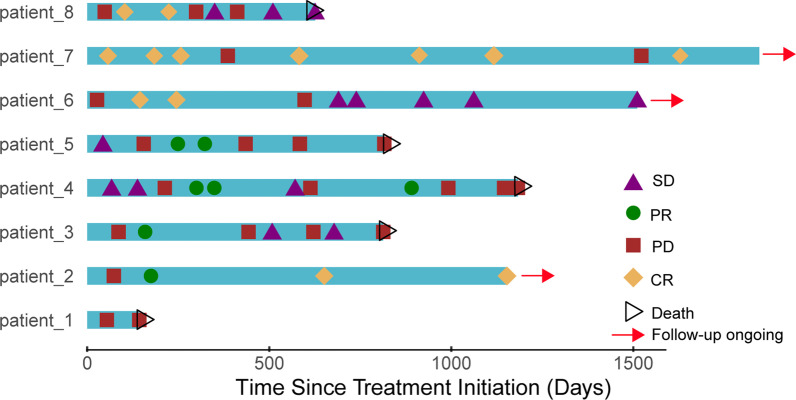
Survival time and tumor efficacy of 8 patients with Nasopharyngeal Carcinoma

**Fig. 5:**
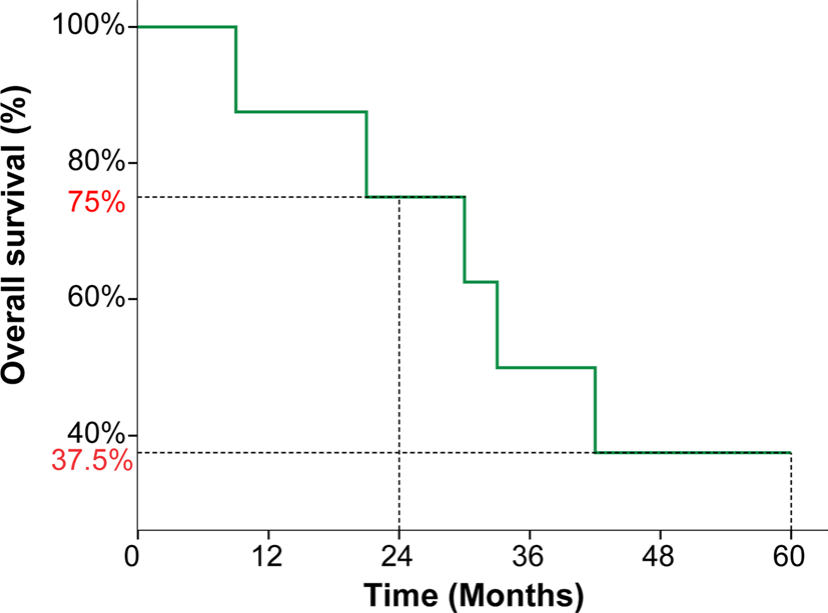
Kaplan–Meier survival curves of OS% in 8 patients with Nasopharyngeal Carcinoma treated with immunotherapy and stereotactic ablative radiotherapy

All included patients received 5–30 doses of PD-1 inhibitors (median: 14 cycles). Figures 3 and 4 show the duration of study treatment for all eight patients, in which evaluation time points and results are clearly detailed. Changes in tumor burden over time, and the specific time of radiotherapy are also shown. For the eight patients, the median OS was 30.9 months 95%CI (15.6, 46.1), and the 2-year OS was 75% (Fig. 5). The PFS of the seven patients who did not respond to PD-1 inhibitors was 10 months, with the 1-year PFS at 43.5%.

### Toxicity of immune-radiotherapy

Seven out of eight patients (87.5%) experienced at least one adverse event. Adverse events that occurred in 15% or more of the patients were as follows; fatigue, asthenia, nausea, decreased appetite, diarrhea, dry mouth, vomiting, rash, pruritus, and anemia (Table 3). Grades 3–4 related toxicity occurred in three cases (37.5%), including one each of nausea, decreased appetite, and vomiting. There were no treatment related dose interruptions and no unexpected toxicities occurred.

**Table 3:** Treatment related toxicities

Treatment-related AE	No. (%) of events	
	Any Grade	Grade 3–4
Any	8(100%)	3(37.5%)
Fatigue	6(75.0%)	0
Asthenia	5(62.5%)	0
Nausea	3(37.5%)	1(12.5%)
Decreased appetite	5(62.5%)	1(12.5%)
Diarrhea	2(25.0%)	0
Dry mouth	5(62.5%)	0
Vomiting	3(37.5%)	1(12.5%)
Rash	7(87.5%)	0
Pruritus	6(75.0%)	0
Anemia	2(25.0%)	0
Pneumonia	1(12.5%)	0
Neutropenia	1(12.5%)	0
Myalgia	0	0
Autoimmune hepatitis	0	0
Dyspnoea	0	0

### Atypical case who achieved complete responses after combined SBRT with PD-1 inhibitors

**Fig. 6:**
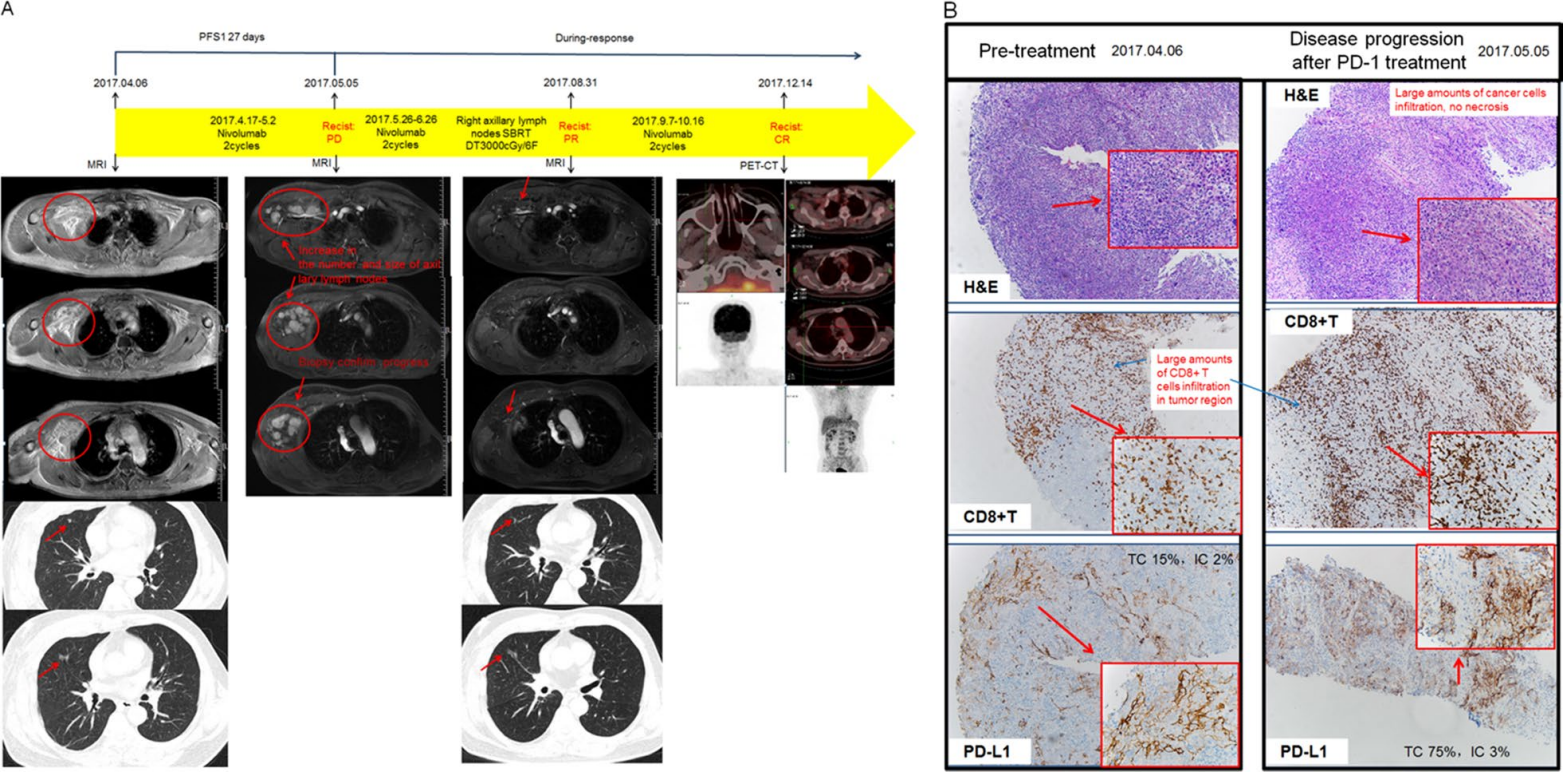
The imaging (**A**) and pathological microenvironmental (**B**) changes of patient No.6 underwent immunotherapy and stereotactic ablative radiotherapy

Patient #6 was a 56-year-old man who had been heavily pretreated and received PD-1 inhibitor as 4th line systemic therapy for metastatic disease in the right 6th rib and right axillary lymph nodes, as showed in Fig. 6. A large number of CD8 + T cells in the tumor region together with PD-L1 positive expression (17%: TC 15%, IC 2%) were observed in a pretreatment biopsy. After two doses of nivolumab, radiologic measurements indicated that lymph nodes in the right axillary were increased in number and size. A subsequent biopsy in this area excluded the possibility of pseudoprogression and showed a similar immune microenvironment to a pre-dose tissue sample. There was no evidence of an existing immune response and no necrosis was observed under the microscope. After discussion by the multidisciplinary treatment group it was decided to combine radiotherapy so as to convert the tumor cells into an in situ tumor vaccine, as indicated in a previous report [21]. He then underwent 3rd and 4th cycles of nivolumab followed by SBRT given in between at all detected metastatic sites (right 6th rib and axillary lymph nodes), 3000 cGy in six fractions were delivered. Five weeks after the SBRT a PET-CT scan showed a PR with smaller metastatic sites in the right 6th rib and axillary lymph nodes. Considering the effectiveness of the combined therapy, another two doses of nivolumab were given. Finally, as presented in Fig. 3, PET-CT imaging showed all metastatic lesions had disappeared, and a metabolic CR was declared.

## Discussion

Although a PD-1/PD-L1 blockade has resulted in impressive clinical responses in some RM-NPC cases, its effectiveness is still far from satisfactory in most patients. Optimal results will require combination with other treatment modalities (immune-modulating treatments), such as SBRT, to enhance a systemic clinical response. The ORR to ICIs alone in our cohort is only 12.5% at first evaluation, while radiation therapy produced an excellent response in PD-1 inhibitor-resistant patients, with the confirmed ORR and CR reported to be of 85.7% and 28.6%. Notably, neither an abscopal effect nor pseudoprogression were observed in our study. Our results are of paramount importance in that this is the first report on the combination of PD-1 inhibitors and radiotherapy in refractory RM-NPC. Our results are consistent with the stimulation of anti-tumor activity by a combination of SBRT with anti-PD-1 treatment and may maximize the clinical benefit of PD-1 inhibitors in this subset of patients.

NPC tumors are characterized by an abundant immune infiltration, the ORR for refractory RM-NPC treated with PD-1 alone, however, was not by the mechanism we expected. As reported in the current study, only an ORR of 12.5% was achieved at first evaluation. This may be a consequence of low tumor mutation burden and limited specific antigens that could be recognized by immune cells that would be insufficient for effectively eliminating tumor cells [37]. Likewise, results of published studies with refractory RM-NPC indicated less than satisfactory, but slightly higher ORR (ranging from 19 to 34%) than ours [13–15]. This might be due to different assessment time point, as all patients underwent their first response evaluation just after 2–3 cycles of PD-1 inhibitors. If PD-1 inhibitors continue to be used alone, the ORR might increase. The retrospective nature and relatively small sample size of our study may be other reasons contributing to this dissimilarity.

The most exciting finding of this study was that radiotherapy can effectively reverse drug-resistance of PD-1 inhibitors. Of the seven patients who were resistant to PD-1 inhibitors, 85.7% (6/7) of them experienced an ORR after receiving radiation, with the median PFS and 2-year OS of 8 months and 71.0%. Indeed, preclinical evidence had clearly indicate that radiotherapy might increase response rates to immune checkpoint inhibitors (ICIs) by creating a more permissive tumor microenvironment [32, 38, 39]. Combining anti-PD-1 treatment with radiotherapy can result in improved clinical response rates and prolong survival [33, 40–42]. However, radiotherapy intervention in those studies were integrated into the treatment plan in advance, either before or during the treatment with ICIs. Masini et al. have reported from the phase II NIVES clinical trial that radiotherapy before ICI therapy did not improve outcome in Renal Cell Carcinoma patients [43]. Whereas in our cohort radiotherapy was given only when PD-1 inhibitor-resistance appeared and was intended to start an immune response by radiation. What was strikingly noticeable was that all irradiated lesions achieved ORR (CR or PR) at second evaluation, but an abscopal effect was not observed in unirradiated lesions.

There has been a systematic review that reported that the mean incident for an distant/abscopal response was 41% in 1736 non-small-cell lung cancer patients treated with an ICIs-SABR combination [44]. However, some investigators argued that this so-called distant response of non-irradiated lesions cannot be called a real abscopal response, since ICIs are systemic broad-spectrum anti-tumor drugs [45]. Thus, these patients may have a systemic anti-tumor effect, even if they were not irradiated. The real abscopal effect should be defined as treatment of ICIs being ineffective, and the irradiation of major or progressive lesions can mediate tumor regression at a remote site. Recently, Elisa Funck-Brentano et al. [45] reported a retrospective study that correctly analyzed the abscopal effect, in which 26 melanoma patients who failed anti-PD-1 monotherapy were included. The abscopal effect was seen in 35% of patients who received hypo-fractionated radiotherapy combined with the anti-PD-1 monoclonal antibody regimen. This surprisingly high incidence of an abscopal effect in melanoma may be explained by the strong immunogenicity of melanoma, owing to which radiotherapy may more easily stimulate an overall immune response.

There are currently two main theories for how radiotherapy activates immunity that are supported by experimental data from animal models. One view is abnormal proteins caused by radiotherapy irradiation play the role of in situ vaccines, generating systemic anti-tumor immunity and an abscopal effect [46]. The other is that broken double-stranded DNA (dsDNA) could be produced in large quantities by irradiated tumor cells that would trigger the cGAS-STING signaling pathway after entering the cytoplasm, and mediate the elimination of damaged tumor cells by immune cells [47]. Results from our study suggested that the latter mechanism may play a greater role. The current study indicated that all irradiated lesions achieved good local control, but no obvious abscopal effect was observed in unirradiated lesions. As Prof. Chang from MD Anderson Cancer Center has proposed, it is time to abandon single-site irradiation for inducing abscopal effects [26]. Perhaps it is necessary for radiation oncologists to rethink how to use our effective weapons (i.e., SBRT) to provide local consolidative therapy (LCT) for all lesions or as many lesions as possible, but solely rely on an abscopal effect to control tumors. Data from phase III trials testing the combination of radiotherapy with ICI have shown that patients who derived benefits either had good prognostic factors and a smaller disease burden (oligometastatic disease) or received irradiation to all sites of gross disease [48, 49]. Furthermore, in melanoma patients the benefit of ICI and the ability to maintain robust antitumor immunity seems to be greatest for patients with a lower total tumor burden [50]. This suggests that a reduction in tumor burden, which could be obtained using comprehensive (but not single site) radiotherapy, could help to potentiate ICI and extend overall survival. Thus, we contend that using comprehensive radiotherapy (i.e., LCT) in combination with ICIs is an important, albeit unexplored, strategy for the optimization of approaches that combine radiotherapy with ICIs.

Published data have confirmed that LCT can effectively improve disease control and overall survival of oligometastasis in various types of cancer [34, 35, 51], its combination with ICI in patients with oligometastasis has shown meaningful clinical outcomes as well [52, 53]. However, there is no relevant report assessing the definite clinical benefit of LCT for ICI in patients with multiple metastases who have more treatment difficulty than oligometastasis. For such a particular subset of patients with a high tumor burden, ICIs alone may not yield a satisfactory clinical response, except for the few cases with a high mutation load or microsatellite instability (MSI). Of note, two multiple metastatic NPC cases who did not response to ICIs alone (patient #2 and #6) achieved an exciting response (CR) after LCT when SBRT was delivered to all visible lesions, suggesting that tumor debulking by LCT could create conditions to ignite local immunity and then enhance tumor response.

In the current study, toxicities associated a PD-1 inhibitor combined with SBRT were generally tolerable, with no lethal toxicity occurring. Grade 3–4 toxicities appeared in 37.5% of patients including nausea, decreased appetite, and vomiting, but did not cause treatment interruption. Our results suggest that this combined modality is feasible and is consistent with data reported by other investigators.

Although this is the first study showing that PD-1 inhibitors combined with SBRT can achieve encouraging results in the treatment of refractory RM-NPC, the retrospective nature and small sample size limits the extensive applicability. Thus, the benefits elicited by this combination are not yet formally established. In addition, additional tumor biology analysis of the ICI-resistant mechanism was not performed in our study and need to be considered in further studies.

## Conclusions

The treatment was well tolerated, with clinical activity supporting the augmentation role of SBRT upon PD-1 inhibitors. Much remains to be learned regarding the optimal dose and/or fractionation schedules of radiotherapy and the mechanism by which SBRT stimulates an immune response. Further well-designed prospective studies are warranted to confirm the complementary role of radiotherapy and PD-1 inhibitors.

## Data Availability

The datasets analyzed during the current study are not publicly available.

## Abbreviations

NPC: Nasopharyngeal carcinoma
RM-NPC: Recurrent/metastatic nasopharyngeal carcinoma
SBRT: Stereotactic body radiation therapy
ISABR: Immunotherapy and stereotactic ablative radiotherapy
ECOG PS: Eastern Cooperative Oncology Group performance status
TIL: Tumor infiltration lymphoma
GTV: Gross Target Volume
PFS: Progression free survival
OS: Overall survival
ORR: Objective response rate
PTV: Planning Target Volume
DCR: Disease control rate
AE: Adverse event
ICIs: Immune checkpoint inhibitors
LCT: Local consolidative therapy
CR: Complete response
